# Gut microbiota, blood metabolites, and left ventricular diastolic dysfunction in US Hispanics/Latinos

**DOI:** 10.1186/s40168-024-01797-x

**Published:** 2024-05-10

**Authors:** Kai Luo, Alkis Taryn, Eun-Hye Moon, Brandilyn A. Peters, Scott D. Solomon, Martha L. Daviglus, Mayank M. Kansal, Bharat Thyagarajan, Marc D. Gellman, Jianwen Cai, Robert D. Burk, Rob Knight, Robert C. Kaplan, Susan Cheng, Carlos J. Rodriguez, Qibin Qi, Bing Yu

**Affiliations:** 1 Department of Epidemiology and Population Health, Albert Einstein College of Medicine, Bronx, NY 10461 USA; 2 Department of Epidemiology, Human Genetics and Environmental Sciences, School of Public Health, University of Texas Health Science Center at Houston, Houston, TX 77030 USA; 3 Cardiovascular Medicine, Brigham and Women’s Hospital, Boston, MA 02115 USA; 4 Institute for Minority Health Research, University of Illinois Chicago College of Medicine, Chicago, IL 60612 USA; 5 Clinical Medicine, University of Illinois College of Medicine, Chicago, IL 60612 USA; 6 Department of Laboratory Medicine & Pathology, University of Minnesota Medical School, Minneapolis, MN 55455 USA; 7 Department of Psychology, Clinical Research Building, Miller School of Medicine, University of Miami, Miami, FL 33136 USA; 8 Department of Biostatistics, The University of North Carolina at Chapel Hill, Chapel Hill, NC 27599 USA; 9 Department of Microbiology and Immunology, Albert Einstein College of Medicine, Bronx, NY 10461 USA; 10 Department of Obstetrics and Gynecology and Women’s Health, Albert Einstein College of Medicine, Bronx, NY 10461 USA; 11 Department of Pediatrics, Albert Einstein College of Medicine, NY10461, Bronx, USA; 12 Center for Microbiome Innovation, University of California, La Jolla, San Diego, CA 92093 USA; 13 Department of Bioengineering, University of California, La Jolla, San Diego, CA 92093 USA; 14 Department of Pediatrics, University of California, La Jolla, San Diego, CA 92093 USA; 15 Department of Computer Science and Engineering, University of California, La Jolla, San Diego, CA 92093 USA; 16 Public Health Sciences Division, Fred Hutchinson Cancer Research Center, Seattle, WA 98109 USA; 17 Smidt Heart Institute, Cedars-Sinai Medical Center, Los Angeles, CA 90048 USA; 18 Department of Medicine, Albert Einstein College of Medicine, Bronx, NY 10461 USA; 19 Department of Nutrition, Harvard T.H. Chan School of Public Health, Boston, MA 02115 USA

**Keywords:** Left ventricular diastolic dysfunction, Gut dysbiosis, Blood metabolome, Hispanic/Latino individuals

## Abstract

**Background:**

Left ventricular diastolic dysfunction (LVDD) is an important precursor of heart failure (HF), but little is known about its relationship with gut dysbiosis and microbial-related metabolites. By leveraging the multi-omics data from the Hispanic Community Health Study/Study of Latinos (HCHS/SOL), a study with population at high burden of LVDD, we aimed to characterize gut microbiota associated with LVDD and identify metabolite signatures of gut dysbiosis and incident LVDD.

**Results:**

We included up to 1996 Hispanic/Latino adults (mean age: 59.4 years; 67.1% female) with comprehensive echocardiography assessments, gut microbiome, and blood metabolome data. LVDD was defined through a composite criterion involving tissue Doppler assessment and left atrial volume index measurements. Among 1996 participants, 916 (45.9%) had prevalent LVDD, and 212 out of 594 participants without LVDD at baseline developed incident LVDD over a median 4.3 years of follow-up. Using multivariable-adjusted analysis of compositions of microbiomes (ANCOM-II) method, we identified 7 out of 512 dominant gut bacterial species (prevalence > 20%) associated with prevalent LVDD (*FDR-q* < 0.1), with inverse associations being found for *Intestinimonas_massiliensis*, *Clostridium_phoceensis*, and *Bacteroide_coprocola* and positive associations for *Gardnerella_vaginali*, *Acidaminococcus_fermentans*, *Pseudomonas_aeruginosa*, and *Necropsobacter_massiliensis*. Using multivariable adjusted linear regression, 220 out of 669 circulating metabolites with detection rate > 75% were associated with the identified LVDD-related bacterial species (*FDR-q* < 0.1), with the majority being linked to *Intestinimonas_massiliensis*, *Clostridium_phoceensis*, and *Acidaminococcus_fermentans*. Furthermore, 46 of these bacteria-associated metabolites, mostly glycerophospholipids, secondary bile acids, and amino acids, were associated with prevalent LVDD (*FDR-q* < 0.1), 21 of which were associated with incident LVDD (relative risk ranging from 0.81 [*p* = 0.001, for guanidinoacetate] to 1.25 [*p* = 9 × 10^−5^, for 1-stearoyl-2-arachidonoyl-GPE (18:0/20:4)]). The inclusion of these 21 bacterial-related metabolites significantly improved the prediction of incident LVDD compared with a traditional risk factor model (the area under the receiver operating characteristic curve [AUC] = 0.73 vs 0.70, *p* = 0.001). Metabolite-based proxy association analyses revealed the inverse associations of *Intestinimonas_massilliensis* and *Clostridium_phoceensis* and the positive association of *Acidaminococcus_fermentans* with incident LVDD.

**Conclusion:**

In this study of US Hispanics/Latinos, we identified multiple gut bacteria and related metabolites linked to LVDD, suggesting their potential roles in this preclinical HF entity.

Video Abstract

**Supplementary Information:**

The online version contains supplementary material available at 10.1186/s40168-024-01797-x.

## Background

Left ventricular diastolic dysfunction (LVDD), characterized by abnormal LV diastolic distensibility, impaired filling, and slow or delayed relaxation, is an important monosymptomatic precursor of heart failure (HF) with a clear progression towards overt stages [1, 2]. The prevalence of LVDD is remarkably high, estimated to be 27~36% in general adults [3–5] but exceeding 60% in aged (> 65 years old) people and individuals with cardiometabolic diseases [6]. Remarkable racial/ethnic differences in LVDD prevalence have been documented. Our previous study showed that compared to other non-Hispanic ethnic groups [4, 7], Hispanic/Latino population has an unexpectedly higher prevalence of LVDD (50.3% among people aged 45 years old or older) [8]. Despite the high prevalence, risk factors for the development of LVDD and underlying mechanisms are not fully understood.

Emerging evidence has linked gut dysbiosis (i.e., unfavorable alterations in the gut microbiota) to clinical HF and its subtypes (e.g., HF with preserved ejection fraction [HFpEF)]) [9–11]. Although the mechanisms underlying the “gut-HF” axis remain largely unknown, the increased filling pressure and impaired diastole that could cause cardiac output to gradually decrease have been proposed as one of the major drivers for gut dysbiosis [11, 12]. Altered gut microbiota composition, such as reduced microbial α-diversity, depleted beneficial bacteria [e.g., the potential short-chain fatty acid (SCFA) producers], and enriched pathogenic bacteria, has been observed in HF patients [10, 11]. Furthermore, it has been shown that gut dysbiosis can affect human health by altering the host circulating metabolite profile as the gut microbiota, both independently and in concert with the host, is capable of producing a diverse range of functional metabolites that can enter the bloodstream [13, 14]. Previous metabolomic studies have found a number of circulating metabolites, including several metabolites related to gut microbiota (e.g., branched-chain amino acids [BCAAs]), associated with LVDD [15–17] and HFpEF [18]. However, to the best of our knowledge, no studies have integrated the gut microbiome and the blood metabolome data with LVDD.

In this study, we leveraged the gut microbiome and blood metabolome data from 1996 adults aged 45 to 75 years who had a comprehensive echocardiographic assessment in the Hispanic Community Health Study/Study of Latinos (HCHS/SOL) to investigate the relationships of gut microbiota and related metabolites with LVDD.

## Methods

### Study design and population

The HCHS/SOL is an ongoing prospective study of Hispanic/Latino population in the USA, with 16415 adults aged 18–74 years recruited in four cities in the United States [19]. The Echocardiographic Study of Latinos (ECHO-SOL) is an ancillary study of the HCHS/SOL designed to characterize the cardiac structure and function in US Hispanic/Latino adults, with 1818 participants aged 45 or over enrolled from 2011 to 2014 (baseline, V1) [8]; among them, 1643 were followed up with cardiac parameters re-examined at the second clinical visit (V2) during 2014–2017 [20]. At V2, the echocardiographic examination was expanded to another independent subset (*N* = 6611) of the HCHS/SOL participants in the echocardiographic reading centers (ECHORC-SOL). In the present study, participants with prevalent cardiovascular diseases (CVD) at baseline were excluded from analyses of LVDD. An overview of study population and study design is shown in Fig. S1 and Supplementary method1. All participants provided written informed consent. The present study was approved by the Institutional Review Board of Albert Einstein College of Medicine, University of Texas Health Science Center at Houston, and University of North Carolina at Chapel Hill.

### Assessment of LVDD

LVDD was defined using multiple echocardiographic parameters measured by a standard transthoracic echocardiography examination as previously described [8], including (1) peak early (E) and late (A) diastolic transmitral inflow velocities, (2) mitral early diastolic (e′) annular velocities and the E/e′, and (3) left atrial volume index (LAVi). As shown in Fig. S2, LVDD was classified into three grades: grade I (mild), grade II (moderate), and grade III (severe). Considering the limited sample size by grade, we combined these three grades as cases and analyzed LVDD as a binary variable in the main association analyses, with those with grade 0 coded as reference (controls). Additional details on the definition of LVDD and exclusion were provided in Supplementary method2.

### Gut microbiome profiling

Metagenomics sequencing was performed on DNA extracted from fecal samples that were collected by Flinders Technology Association (FTA) cards [21, 22] from 3035 participants who were enrolled in the Gut Origins of Latino Diabetes (GOLD), the ancillary microbiome study of the HCHS/SOL at V2 [21–23]. SHOGUN pipeline was used to profile microbiome taxonomic and functional features [24]. We included 1996 participants with both echocardiography assessments and gut microbiome data (1508 were from ECHORC-SOL and 488 were from ECHO-SOL at V2) in the present study. Gut bacterial species presented in more than 20% of samples were included in the association analyses, in which the abundances of species were centered log-ratio (CLR) transformed. Additional information was provided in Supplementary method3.

### Serum metabolome profiling

Serum metabolomics was conducted using the discoveryHD4 platform at Metabolon (Durham, NC, USA) [25] in 6180 participants at baseline and 814 participants who participated in the GOLD at V2. A total of 669 known metabolites detected in > 75% of participants were included, whose levels were rank-based inverse normal transformed (INT) after replacing levels below detection by half of the minimum detected value. Additional information was provided in Supplementary method4.

### Statistical analysis

To identify gut microbial species associated with prevalent LVDD, we first conducted discovery analyses among 1508 participants (658 prevalent LVDD cases) in the ECHORC-SOL at V2 (i.e., discovery set) and then replicated the associations in 488 participants (258 prevalent LVDD cases) from the ECHO-SOL at V2 (i.e., validation set) (Fig. S1). Analysis of compositions of microbiomes (ANCOM-II) method was applied to identify bacterial species associated with prevalent LVDD in the discovery stage, in which false discovery rate (FDR) was controlled at 0.1 and estimates were adjusted for age, sex, study center, and usage of antibiotics or probiotics. ANCOM-II is an improved method for microbial difference analysis, which can account for compositional structure and the type of zeros (e.g., outlier, structural, and sampling zeros) in the microbiome data, when comparing the abundance of taxa to a background value (e.g., the background value of a specific taxa the users specified or a value specific to each specimen like the geometric mean) rather than the overall taxa abundance in the ecosystem of compared groups, an approach adopted in its predecessor ANCOM [26, 27]. In addition, both ANCOM-II and ANCOM are able to perform microbial difference analysis in a linear model framework with adjustment for covariates [26, 27]. The quantitative associations of species selected by ANCOM-II with prevalent LVDD were assessed using binary logistic regression in both discovery and validation sets. A random-effect meta-analysis was further performed to pool estimates from the above analytical sets. Species with significant pooled results (*p* < 0.05) and consistent directionality in the two analytical sets were retained, and the final associations of these species with LVDD were assessed in all participants (*N* = 1996) using binary logistic regression models adjusted for age, sex, study center, education, annual household income, smoking status, alcohol consumption status, physical activity, alternative healthy eating index 2010 (i.e., AHEI2010 for overall dietary quality [28]), and usage of antibiotics or probiotics at the time of stool samples collection (Model1). The robustness of associations between bacterial species and prevalent LVDD was evaluated by further adjusting for body mass index (BMI) and systolic blood pressure (SBP) based on Model1 (Model2) and adjusting for the use of antidiabetic, antihypertensive, and lipid lowering medications based on Model2 (Model3). Details on the definitions of these covariates were provided in the Supplementary method5 and Table S1.

To identify metabolites associated with prevalent LVDD-associated bacterial species, metabolome-wide association analyses (MWAA) were conducted among 804 participants with concurrent gut microbiome and metabolome data using linear regression models. The associations of species-associated metabolites with prevalent LVDD were then examined among 1405 participants (695 prevalent cases) enrolled in the ECHO-SOL at V1, using binary logistic regression models. FDR was controlled at 0.1 for these analyses. We further conducted a prospective analysis among 594 participants who were free of LVDD at baseline to assess the associations between the identified prevalent LVDD-associated metabolites with incident LVDD (212 incident cases were identified over a median 4.3 years of follow-up) using a modified Poisson regression, a method designed to assess relative risk (RR) for common outcomes (e.g., proportion of incident cases > 10%) in prospective analysis [29]. Covariates used in the Model1 were adjusted in these association analyses. To assess relative importance of identified metabolites and traditional risk factors (age, BMI, SBP, smoking, drinking, low low-density lipoprotein cholesterol (LDL-C), and diabetes) in predicting incident LVDD, we performed an elastic net regression (ENR) analysis with a 10 × 10-fold nested cross-validation (nestedcv) [30] and calculated the SHapley Additive exPlanations (SHAP) values and area under the receiver operating characteristic (ROC) curve (AUC).

Microbiome functional analyses based on KEGG Orthology (KO) were also conducted to interpret the observed associations between LVDD-associated bacteria species and metabolites. A total of 2042 KOs presented in > 20% of samples with at least 0.001% of relative abundance were included. The associations of KOs with species and metabolites were assessed among 804 participants with both metabolomics and metagenomics data using linear regression, while their associations with prevalent LVDD were assessed among 1996 participants with metagenomic and echocardiographic data using binary logistic regression. The intercorrelation among species, KOs, and metabolites was assessed through partial correlation analyses. Covariates in the above mentioned Model1 in analyses of bacteria species and prevalent LVDD were adjusted in these regression and partial correlation analyses. FDR was controlled at 0.1.

Given the lack of prospective data for the gut microbiome and incident LVDD analysis, we conducted “proxy association” analyses [31] by using identified bacterial-associated metabolites as proxies to indirectly assess the relationship between gut bacteria and incident LVDD. In brief, the effect estimates for the associations of metabolites with bacteria species and incident LVDD were standardized into Z-scores (i.e., the ratios of beta coefficients to their respective standard errors). A spearman correlation was then conducted to link Z-scores from metabolites-species associations with those from metabolites-LVDD associations, in which a correlation *p*-value < 0.05 indicated a significant proxy association between gut bacterial species and incident LVDD. Only metabolites significantly associated with incident LVDD were included in this proxy-association analysis.

All analyses were conducted via R 4.1. Additional information on these analytical models and corresponding R packages is provided in Supplementary method5.

## Results

### Population characteristics

Table S1 described the population characteristics by LVDD status among 1996 participants (1508 in ECHORC-SOL; 488 in ECHO-SOL) with both echocardiographic measurements and gut microbiome data at V2 in the HCHS/SOL. Main characteristics were generally similar between two datasets. Overall, individuals with LVDD tended to be older, female, and less physically active; have a higher education, BMI, and SBP; and be more likely to take antihypertensive, antidiabetic, and lipid-lowering medications compared to non-LVDD individuals.

### Gut microbial species associated with prevalent LVDD

**Fig. 1:**
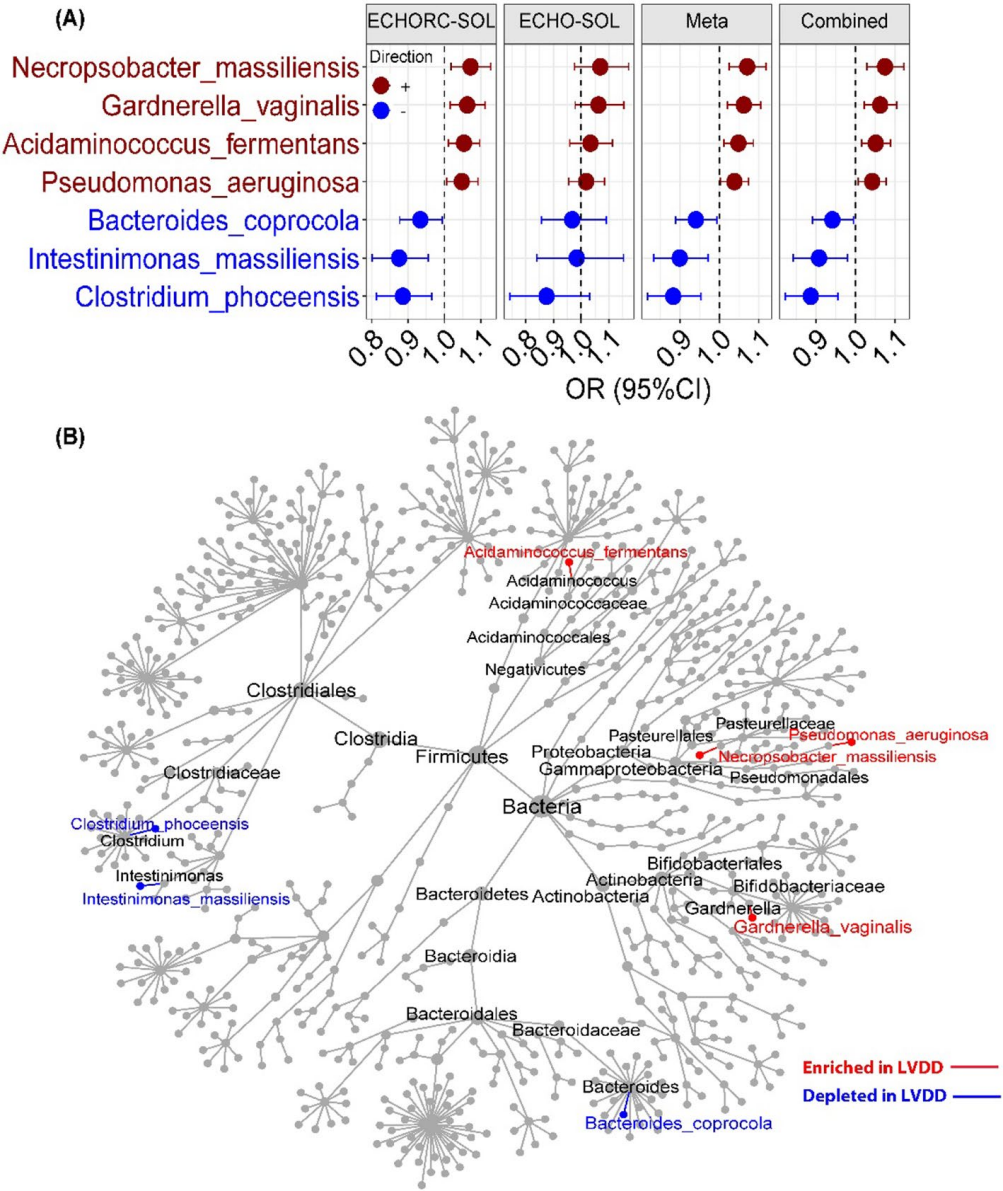
Gut microbial species and prevalent left ventricular diastolic dysfunction (LVDD). **A** The associations of selected bacteria species with prevalent LVDD in ECHORC-SOL (discovery set, *N* = 1508), ECHO-SOL (validation set, *N* = 488), and combined cohort (*N* = 1996). Data were shown as odds ratios (OR) and 95% confidence interval (CI) from binary logistic regression adjusting for age, sex, study center, education, annual household income, smoking status, alcohol consumption status, physical activity (METs), Alternative Healthy Eating Index 2010 (AHEI2010), and use of antibiotics or probiotics at the time of stool sample collection (Model1). **B** Phylogenetic tree of the selected gut bacterial species associated with prevalent LVDD. Species highlighted in red refer to those enriched in LVDD (i.e., having positive association with prevalent LVDD), while species in blue refers to those depleted in LVDD

Nine bacterial species were identified to be associated with prevalent LVDD in the discovery set (*FDR* < 0.1, Fig. S3), seven of which showed directionally concordant associations with LVDD in both discovery and validation sets (Fig. 1A and Fig. S3). The associations between these seven bacterial species and prevalent LVDD pooled by meta-analysis of these two datasets were identical to those estimated from the combined dataset with both ECHORC-SOL and ECHO-SOL participants (all *p* < 0.05; Fig. 1A). Of those seven species, two members in *Clostridia* class (*Intestinimonas_massiliensis* and *Clostridium_phoceensis*) and *Bacteroides_coprocola* were inversely associated with prevalent LVDD, while positive associations were found for remaining four species (*Gardnerella_vaginali*, *Acidaminococcus_fermentans)* and two members of *Proteobacteria* phylum (*Pseudomonas_aeruginosa* and *Necropsobacter_massiliensis*) (Fig. 1B). We also found a significant linear trend of abundances of these seven species across LVDD grades (Fig. S4). Further adjusting for BMI, SBP, and the use of lipids lowering, antihypertensive, and antidiabetic medications did not materially alter associations of these species with prevalent LVDD (Model2 to Model3 in Table S2).

### Metabolite signatures of LVDD-associated gut bacteria species and their associations with LVDD

**Fig. 2:**
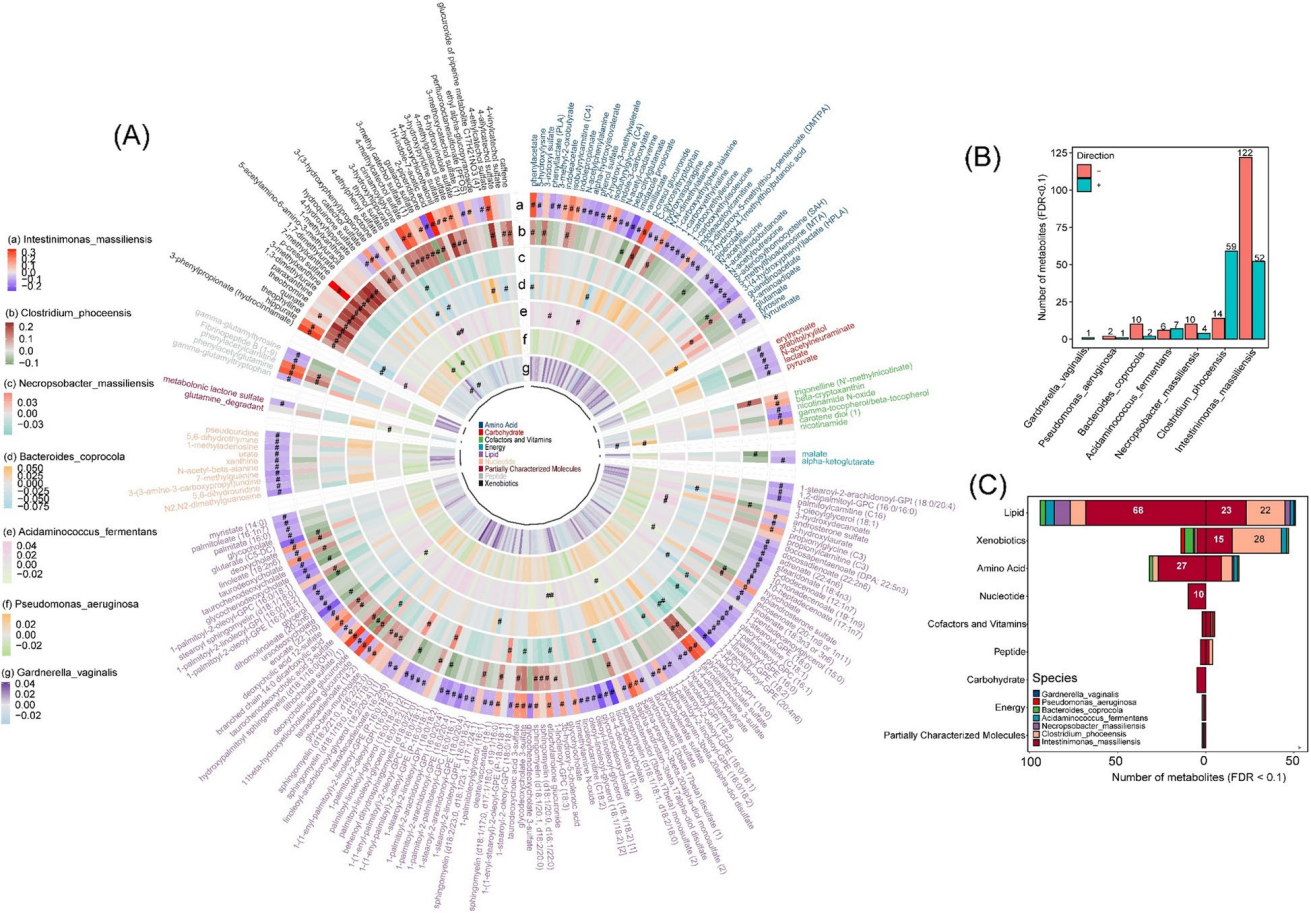
Circulating metabolite signatures of prevalent left ventricular diastolic dysfunction (LVDD)-associated bacterial species. **A** Associations of 220 metabolites with 7 prevalent LVDD-associated bacteria species. Data were shown as the coefficients from multiple linear regression adjusting for age, sex, study center, education, annual household income, smoking status, alcohol consumption status, physical activity (METs), AHEI2010, and use of antibiotics or probiotics at the time of stool sample collection. Only metabolites significantly (*FDR* < 0.1) associated with at least one species were presented here. #*FDR-q* < 0.1. **B** Numbers of significant metabolites associated with each species. **C** The composition of bacterial species-associated metabolites by super pathway. Numbers toward left refer to the numbers of metabolites inversely associated with species, whereas those toward right refer to number of metabolites positively associated with species

**Fig. 3:**
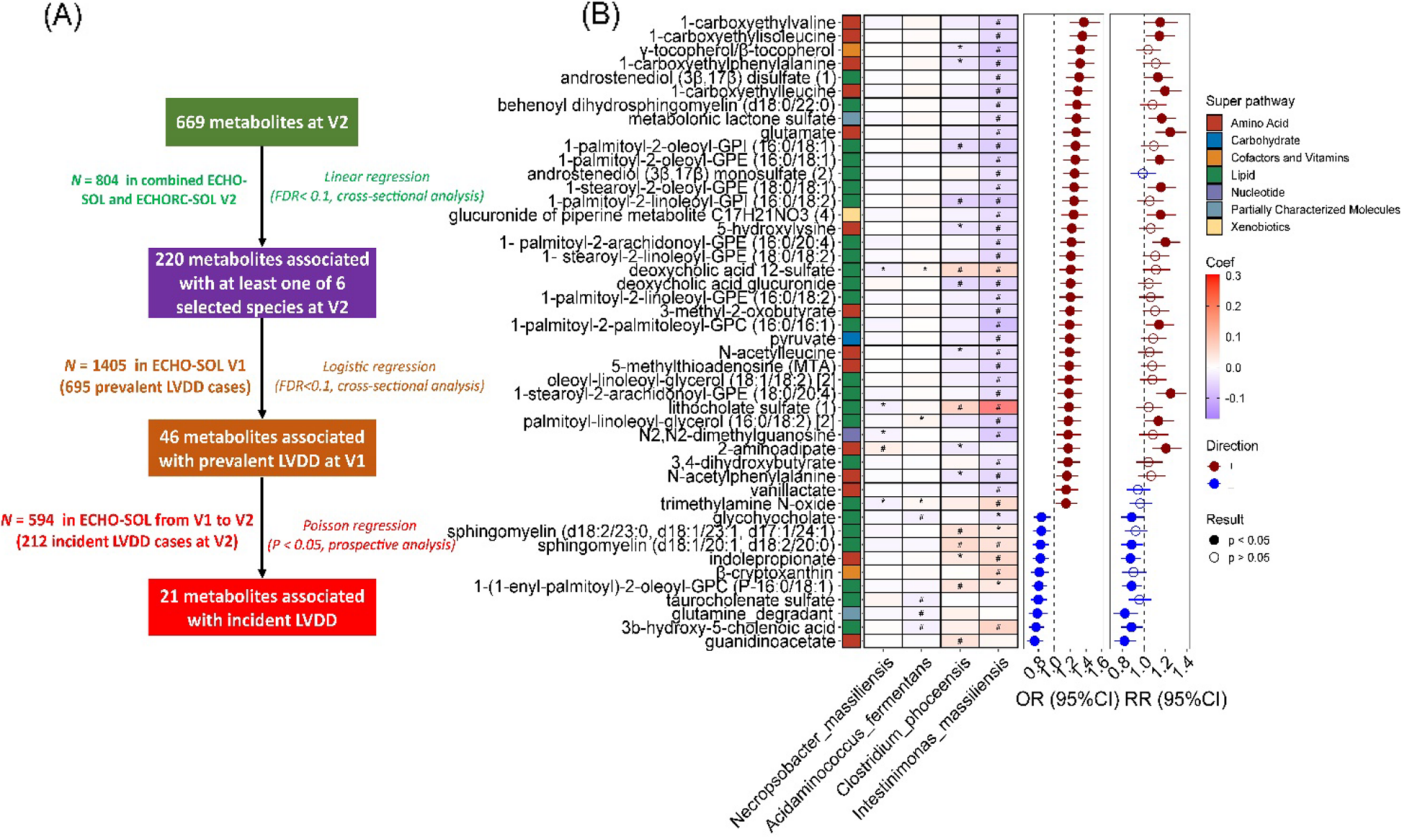
Associations of gut bacterial species-associated metabolites with prevalent and incident left ventricular diastolic dysfunction (LVDD). **A** A summary of analyses for the selection metabolites signature of prevalent and incident LVDD. **B** Association of 46 metabolites associated with at least one of 4 species with the prevalent (the middle forest plot) and incident (the right forest plot) LVDD. The heatmap in the left panel shows associations (coefficients) of four bacterial species with metabolites, adjusting for age, sex, study center, use of antibiotics or probiotics, education, annual household income, smoking status, alcohol consumption status, physical activity (METs), and AHEI2010. #*FDR* < 0.1, *p value < 0.05. The forest plot in the middle panel shows the odds ratios (OR) and 95% confidential intervals (CIs) for associations of 46 metabolites with prevalent LVDD in ECHO-SOL at V1 (*FDR* < 0.1), and the right forest plot panel shows the relative risk ratio (RRs) and 95% CIs of these metabolites with incident LVDD. Estimates were derived from the binary logistic regression and Poisson regression, respectively, adjusting for the same abovementioned covariates, except for the use of antibiotics and probiotics

MWAA identified 220 out of 669 plasma metabolites significantly (*FDR* < 0.1) associated with at least 1 of the 7 LVDD-associated bacterial species (Fig. 2A). Most significant species-metabolite associations were found in *Intestinimonas_massiliensis* (*n* = 174 with 122 showed positive associations) and *Clostridium_phoceensis* (*n* = 73 with 59 showed positive associations), the 2 species inversely associated with LVDD (Fig. 2B). The majority of the associated metabolites were lipids, xenobiotics, and amino acids (Fig. 2C). Furthermore, 46 of those 220 metabolites were significantly associated with prevalent LVDD (*FDR* < 0.1, Tables S3–4 and Fig. 3A). Inverse associations were observed for 10 metabolites, which were led by guanidinoacetate ([odds ratio [OR] = 0.75, 95% confidence interval *CI*: 0.66, 0.86] per 1 SD increase), and positive associations were observed for the remaining 36 metabolites, which were led by 1-carboxyethylvaline [*OR* = 1.37, 95% *CI*: 1.19, 1.58] (middle forest plot in Fig. 3B). Most of these 46 metabolites were associated with *Intestinimonas_massiliensis* (39 metabolites) and *Clostridium_phoceensis* (9 metabolites), the aforementioned 2 prevalent LVDD-depleted species (heatmap in Fig. 3B).

Among these 46 metabolites, 21 were significantly (*p* < 0.05) associated with incident LVDD (Fig. 3A–B and Table S5). Inverse associations with incident LVDD were found for seven metabolites, including two amino acid metabolites (indolepropionate and guanidinoacetate), four lipids (1-(1-enyl-palmitoyl)-2-oleoyl-GPC (P-16:0/18:1), sphingomyelin (d18:1/20:1, d18:2/20:0), and two secondary bile acids [glycohyocholate and 3b-hydroxy-5-cholenoic acid]), and glutamine degradant, while positive associations were found for the remaining 14 metabolites, including three BCAA metabolites (e.g., 1-carboxyethylleucine), five glycerophospholipids (e.g., 1-stearoyl-2-arachidonoyl-GPE (18:0/20:4)), and two xenobiotics or partially characterized molecules (e.g., glucuronide of piperine metabolite C17H21NO3(4) and metabolonic lactone sulfate). The associations of these metabolites with prevalent and incident LVDD were slightly attenuated after further adjustment of BMI, SBP, and use of antidiabetic, lipid-lowering, and antihypertensive medications (Fig. S5 and Tables S4–5).

### Proxy associations between gut bacteria and incident LVDD

**Fig. 4:**
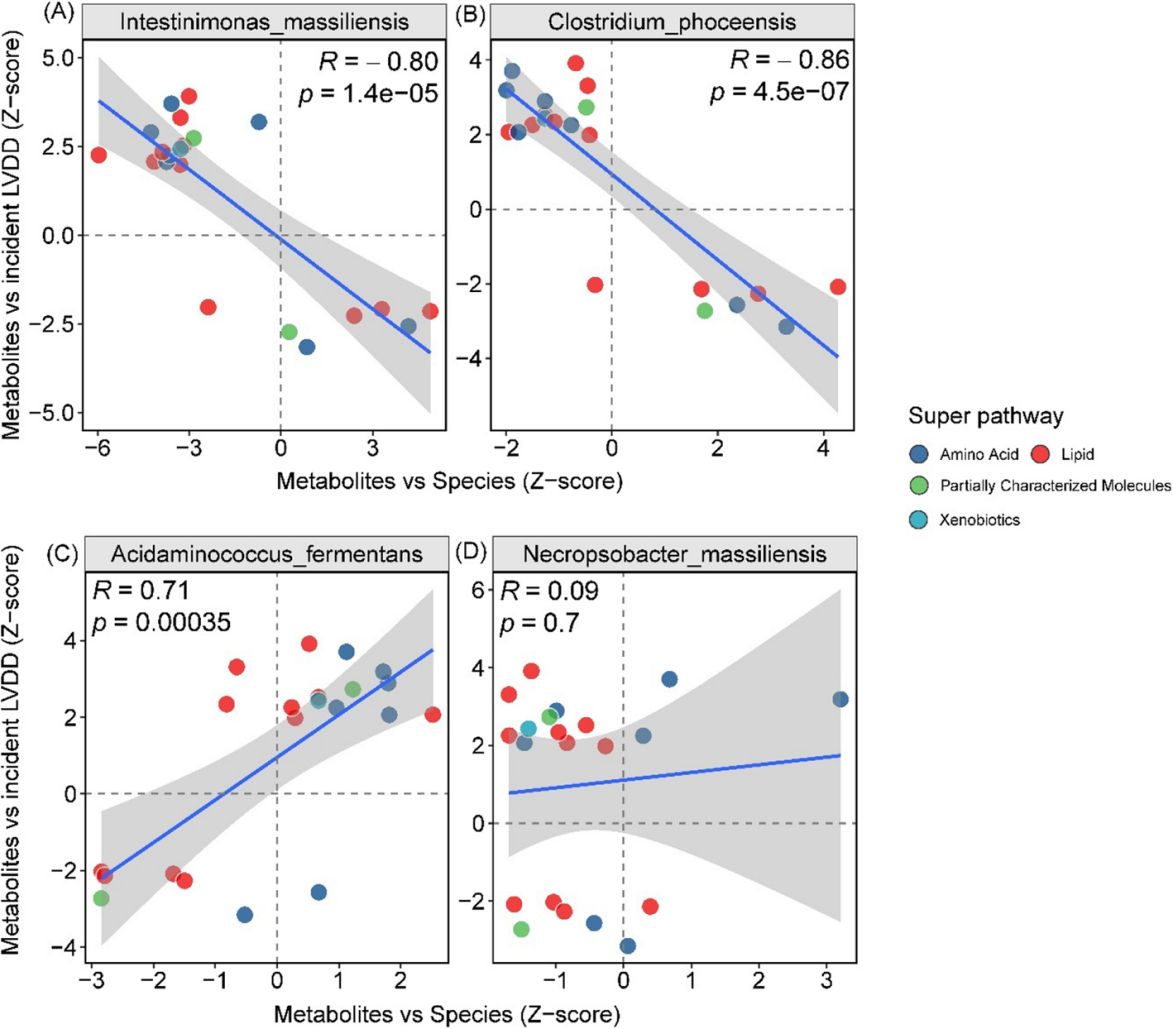
Proxy associations between gut bacterial species and incident left ventricular diastolic dysfunction (LVDD) utilizing 21 metabolites associated with incident LVDD. Metabolite signatures. Each dot represents a metabolite. The *x*-axis shows the Z-scores from associations of metabolites with bacterial species, while the *y*-axis shows Z-scores from associations of metabolites with incident LVDD. Colors of dots represent the super pathways of metabolites

Proxy association analyses utilizing all 21 metabolites that were associated with incident LVDD and bacteria species revealed significant putative inverse associations of *Intestinimonas_massiliensis* (Spearman correlation *R* = −0.80, *p* = 1.4 × 10^−5^) and *Clostridium_phnoceensis* (*R* = −0.86, *p* = 4.5 × 10^−7^) with incident LVDD, whereas a positive correlation was found for *Acidaminococcus_fermentans* (*R* = 0.75 and *p* = 0.00035) (Fig. 4). These results suggested potential prospective associations between these gut bacterial species, indicated by related metabolites as proxies, and incident LVDD.

### Risk prediction of incident LVDD

**Fig. 5:**
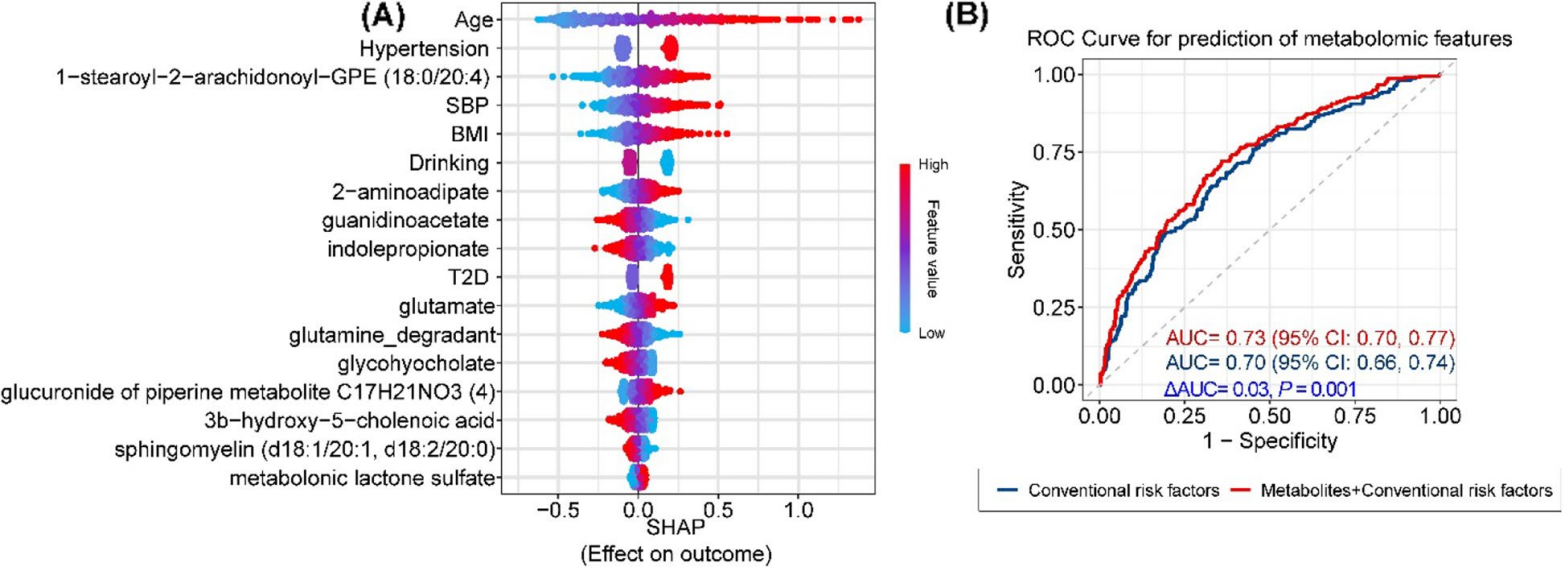
Prediction of incident left ventricular diastolic dysfunction (LVDD) utilizing identified gut bacterial-associated metabolites. **A** Relative importance of selected gut bacterial-associated metabolites and traditional risk factors of heart failure in prediction of LVDD. Data are the SHapley Additive exPlanations (SHAP) values of each included feature. **B** Receiver operating characteristic (ROC) curves of models with only traditional risk factors and additional inclusion of identified bacterial species-associated metabolites. Area under the curve (AUC) was calculated to assess the reclassification performance of the prediction models. The 95% CIs of AUC were estimated via bootstrapping with 10,000 iterations. Details on this prediction analysis were provided in the Supplementary method5

To assess the ability of bacteria-associated metabolites for prediction of incident LVDD beyond traditional risk factors, we compared the ROC curves for the prediction models with and without inclusion of these identified metabolites. Eleven out of the 21 metabolites associated with incident LVDD, along with 5 traditional risk factors (age, SBP, BMI, drinking, and diabetes), were selected to be predictive of incident LVDD during feature selection in the ENR analyses (Fig. 5A). The inclusion of these 11 metabolites for prediction significantly increased the AUC to 0.73 (95% *CI*: 0.70, 0.77) from 0.70 (95% *CI*:0.66, 0.74) based on only selected traditional risk factors (*p* for change in *AUC*: 0.001) (Fig. 5B).

### Integration of gut microbial function, LVDD-associated species, and metabolites

**Fig. 6:**
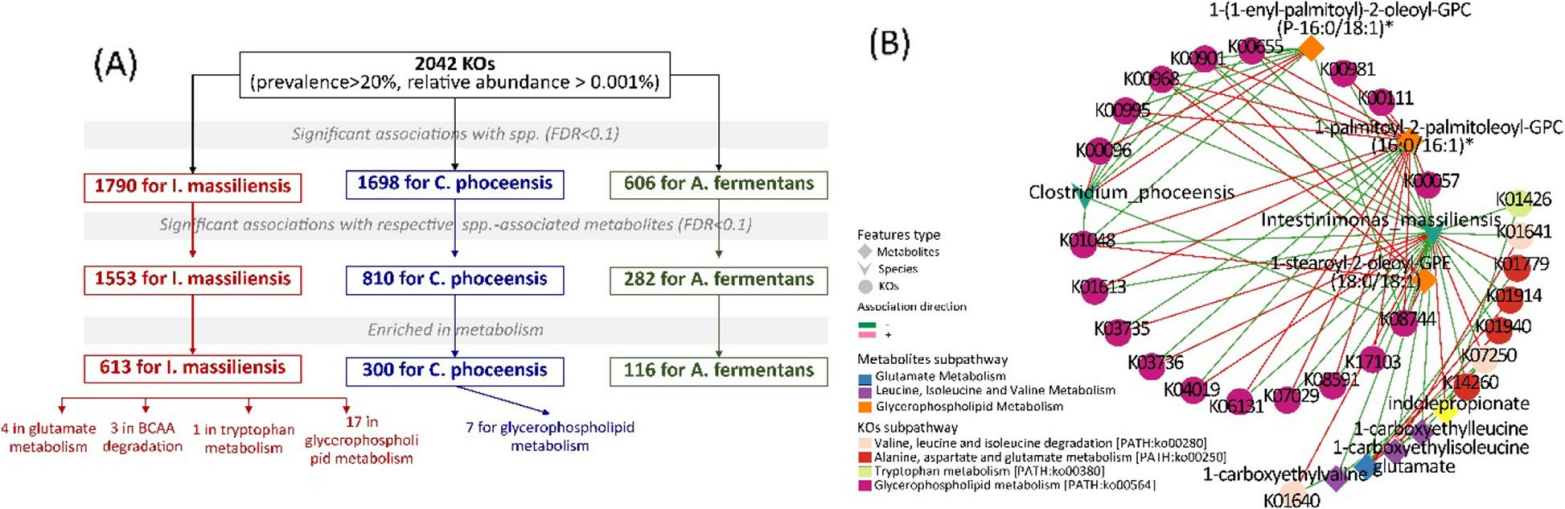
Relationship among identified LVDD-associated species, KEGG Orthology (KOs), and metabolites. A An overview of localization of KOs potentially involved in the metabolism of metabolites that were associated with incident LVDD. *A. fermentans* for *Acidaminococcus_fermentans*; *I. massiliensis* for *Intestinimonas_massiliensis*, and *C. phoceensis* for *Clostridium_phoceensis*. B Interrelationship among LVDD-associated species, KOs concordantly associated with both species and metabolites, metabolites associated with incident LVDD. Edges represent the association coefficients among species, metabolites, and KOs, with those highlighted in red represented for positive association and “blue” for negative association. All association coefficients with *FDR-q* < 0.1 were derived from multivariable regression analyses (logistic model for binary outcomes and linear regression for continuous outcomes) with the adjustment of age, sex, study center, use of antibiotics or probiotics, education, annual household income, smoking status, alcohol consumption status, physical activity (METs), and AHEI2010

Given that only *Inestinimonas_massiliensis*, *Clostridium_phnoceensis*, and *Acidaminococcus_fermentans* exhibited significant proxy associations with incident LVDD, we thus focused on these three species and investigated functional alterations at KO gene level to reveal the observed species-metabolite associations. Using multivariable adjusted linear regression (Model1), we found that a total of 630 unique KOs enriched in metabolism pathways were associated with at least one of these three species and their respective associated metabolites (613 KOs for *Inestinimonas_massiliensis*, 300 for *Clostridium_phnoceensis*, and 116 *Acidaminococcus_fermentans*, all with *FDR* < 0.1) (Fig. 6A and Fig. S7). Among these 630 unique KOs, 47 were nominally associated with LVDD (*p* < 0.05), with the majority showing inverse associations and being positively associated with *Inestinimonas_massiliensis* or *Clostridium_phnoceensis* (Table S6). Most of these 47 KOs were involved in pathways related to propanoate metabolism, oxidative phosphorylation, pyrimidine metabolism, pyruvate metabolism, and purine metabolism (Table S6). We then focused on KOs involved in amino acid and lipid metabolism, the two sub-pathways where the majority of 21 incident LVDD-associated metabolites resided, and found that a total of 31 *Inestinimonas_massiliensis-*associated KOs (Fig. 6B and Table S7) were involved in amino acid and lipid metabolism, including 17 in glycerophospholipid metabolism, four in glutamate metabolism, three in BCAA degradation, one in tryptophan metabolism, and seven *Clostridium_phnoceensis*-associated KOs in glycerophospholipid metabolism (Fig. 6A). Overall, KOs involved in the same metabolism sub-pathway were positively correlated with each other (Fig. S7), having concordant triangular relationships with species and metabolites (Fig. 6B). For example, KOs in glycerophospholipid metabolism that were inversely associated with *Inestinimonas_massiliensis* or *Clostridium_phnoceensis* were also positively associated with species-depleted glycerophospholipids (e.g., 1-palmitoyl-2-palmitoleoyl-GPC (16:0/16:1) and 1-stearoyl-2-oleoyl-GPE (18:0/18:1)). One of such KOs was K01048, the *pldB* gene encoding lysophospholipase [EC:3.1.1.5], participating in the conversion of phosphatidylethanolamine/phosphatidylcholine to sn-glycero3-phosphoethanolamine/sn-glycero-3-phosphocholine (Table S7).

## Discussion

In this study of US Hispanics/Latinos, a population with a high burden of LVDD, we identified seven gut bacterial species associated with prevalent LVDD, and their associations were generally independent of known HF risk factors (e.g., obesity, diabetes, and hypertension). By leveraging circulating metabolomics data, we identified 46 bacterial species-associated metabolites associated with prevalent LVDD, 21 of which were also associated with incident LVDD. The majority of the identified metabolites belonged to glycerophospholipids, secondary bile acids, and BCCA derivatives, which were partially reflected by altered microbial KO abundances involved in metabolism pathways of these metabolites. Findings from the proxy association and risk prediction analyses collectively highlighted the importance of these metabolites, especially those of microbial origin, in reflecting gut dysbiosis, understanding the molecular pathway involved in the development of LVDD, and predicting future risk of this important HF precursor.

Despite the growing body of evidence linking gut dysbiosis to HF [10, 11], little is known about the relationship between gut microbiota and LVDD. One previous study has explored this relationship, in which lower abundances of phylum Bacteroidetes and genera *Bacteroides* were associated with LVDD and poorer echocardiographic parameters [32]. Partially aligning with these findings, we observed an inverse association between *Bacteroides_coprocola* and LVDD. Our data further indicated inverse associations of *Intestinimonas_massilliensis* and *Clostridium_phoceensis*, the two potential beneficial species in *Clostridiales* class, with LVDD. Of note, these two species and many *Clostridiales* members are known nondigestible carbohydrate degraders and SCFA producers [33, 34] that have been reported to be depleted in HF [35], atrial fibrillation [36], and atherosclerotic CVD [37, 38]. Our study also identified four gut bacterial species positively associated with LVDD, including *Necropsobacter_massiliensis*, *Pseudomonas_aeruginosa*, *Gardnerella_vaginalis*, and *Acidaminococcus_fermentans*. Elevated abundances of gut *Pseudomonas_aeruginosa* have also been found in HFpEF patients [39]. In addition, previous studies have reported the presence of *Necropsobacter_massiliensis*, *Gardnerella_vaginalis*, and *Pseudomonas_aeruginosa* as opportunistic pathogenic bacteria in the samples isolated from cervical abscess [40], bacterial vaginosis [41], and infective endocarditis [42], respectively. These findings imply that the perturbation in the gut microbiome, such as depletion in certain beneficial bacteria involved in complex carbohydrate fermentation and enrichment of pathogenic bacteria, may already exist in the early stages of HF.

Our study extends previous investigations on metabolite signatures of LVDD and other cardiac dysfunction [15–17] by highlighting the potential involvement of gut microbiota. In particular, we identified that five glycerophospholipids with palmitoyl or stearoyl group at the sn-1 position of phosphatidylethanolamine (e.g., 1-stearoyl-2-arachidonoyl-GPE (18:0/20:4)) and phosphatidylcholine (e.g., 1-palmitoyl-2-palmitoleoyl-GPC (16:0/16:1)) were inversely associated with *Intestinimonas_massilliensis* and positively associated with incident LVDD. In support of our findings, elevated levels of these glycerophospholipids have been documented in ischemic heart disease [37, 43]. These glycerophospholipids have been found to be inversely associated with *Intestinimonas_massilliensis* and other *Intestinimonas* spp. (e.g., *Intestinibacter_bartlettii*) in a recent study among Swedish individuals [44]. Our integrated analyses with microbial KO genes further suggest that the observed inverse associations between *Intestinimonas_massilliensis* and glycerophospholipids could be partially explained by the inverse association of *Intestinimonas_massilliensis* with abundance of *pldB* (K01048), the key gene encoding the hydrolase lysophospholipase [EC:3.1.1.5] that regulates the biosynthesis of glycerophospholipids with palmitoyl or stearoyl group at sn-1 or sn-2 position. Furthermore, we found that sphingomyelin (sphingomyelin (d18:1/20:1, d18:2/20:0)) and plasmalogen (1-(1-enyl-palmitoyl)-2-oleoyl-GPC (P-16:0/18:1)) that were positively associated with *Clostridium_phoceensis* tended to have protective effect on LVDD. Consistent with our findings, reduced levels of these two lipids have been found in ischemic heart disease [37], and many *Clostridium* members have been linked with elevated levels of numerous plasmalogen and sphingomyelins [44, 45]. Two secondary bile acids (glycohyocholate and 3β-hydroxy-5-cholenoic acid) that were positively associated with *Acidaminococcus_fermentans* have also been found to be inversely associated with incident LVDD in the present study, but the role of these secondary bile acids in LVDD and their relationship with gut microbiota require further investigation.

In addition to lipids, our study identified several metabolites of amino acids, whose metabolism were known to be partially regulated by the gut microbiota, associated with incident LVDD. One such finding is that indolepropionate, a known microbially derived metabolite from tryptophan metabolism [46], was inversely associated with the risk of LVDD and was positively associated with both *Intestinimonas_massilliensis* and *Clostridium_phoceensis*. These findings are supported by the reported cardiometabolic protective effect of indolepropionate [47, 48] and the documented involvement of identified species, especially *Clostridium_phoceensis*, in producing indolepropionate [49, 50]. Interestingly, we found that *Clostridium_phoceensis* was inversely associated with K01426, the gene encoding the amidase to further degrade indole-3-acetamine along the tryptophan-tryptamine pathway (Table S7)*.* We thus suspect that *Clostridium_phoceensis* could directly or indirectly suppress the activity of K01426, potentially shifting the tryptophan catabolism toward pyruvate pathway, enhancing the production of indolepropionate, which could partially explain our observation. Additionally, our study revealed that three BCAA metabolites (1-carboxyethylisoleucine, 1-carboxyethylvaline, 1-carboxyethylleucine) were associated with an increased risk of LVDD. In line with our observations, these circulating BCAA metabolites have been found to be top predictors of microbial α-diversity [51] and to be inversely correlated with several *Intestinimonas* spp., including *Intestinimonas_massilliensis* [44]*.* Taken together, our results provided additional evidence highlighting the potential interplay between gut microbiota and BCAA metabolism in relation to the risk of LVDD.

Our study also found a group of partially characterized molecules (glutamine degradant and metabolonic lactone sulfate), xenobiotics (glucuronide of piperine metabolite C17H21NO3 (4)), and derivatives of nonessential amino acids (glutamate, 2-aminoadipate, and guanidinoacetate) associated with incident LVDD. These findings were partly aligned with studies reporting associations of these metabolites with elevated blood pressure (glucuronide of piperine metabolite C17H21NO3 (4) for both SBP and DBP [52]), increased risk of CVD (e.g., glutamate [53] and metabolonic lactone sulfate [54]), and diabetes (2-aminoadipate [55]). Although direct evidence on the link between the gut microbiota and these metabolites is still lacking, the observed inverse associations of the majority of these metabolites with *Intestinimonas_massilliensis* or *Clostridium_phoceensi*, the two bacterial species inversely associated with LVDD, suggest potentially novel metabolite-related pathways through which these beneficial bacteria may influence LVDD and later the development of HF.

The major strengths of this study include a comparatively large sample size of a US minority population with a high burden of LVDD, comprehensive echocardiography assessment, and both gut microbiome and blood metabolome data. Nevertheless, our study has several limitations. The associations between gut microbiota and LVDD were only examined cross-sectionally. However, we examined the prospective associations between microbiota-related metabolites and incident LVDD as well as conducted proxy association analyses by using identified microbial metabolites as proxies to explore the relationship between gut microbiota and the risk of LVDD. It is crucial to note that our findings are observational in nature, which requires further experimental validations to assess causality. Finally, although we found a statistically significant improvement in the predictiveness of incident LVDD based on the identified bacteria-associated metabolites beyond the conventional risk factors, the increment was modest; thus, the clinical implication of this finding might be limited. Future studies are needed to identify additional novel metabolites associated with incident LVDD.

## Conclusion

In this study of US Hispanics/Latinos, we identified several gut microbial species associated with LVDD, including two potential beneficial bacteria in the Clostridiales order *Intestinimonas_massilliensis* and *Clostridium_phoceensis*. Multiple circulating metabolites were associated with these two beneficial bacteria and incident LVDD during follow-up. Our findings revealed alterations in the gut microbiota and blood metabolome in the early stage of HF, though how these alterations may contribute to the development of overt HF in the future need further investigation.

### Supplementary Information

** Additional file 1:** Supplementary methods: **Supplementary Method 1.** Study design and population. **Supplementary Method 2.** Assessment of LVDD. **Supplementary Method 3.** Gut microbiome profiling. **Supplementary Method 4**. Metabolome profiling. **Supplementary Method 5**. Statistical analysis. Supplementary figures: **Fig. S1.** Overview of the study design and main analyses. **Fig. S2. **Algorithm for assessing left ventricular diastolic dysfunction (LVDD). **Fig. S3.** Pooled results of associations between 9 species selected by ANCOM-II and prevalent LVDD in both discovery (ECHORC-SOL) and validation (ECHO-SOL) sets. **Fig. S4.** Abundances of the identified LVDD-associated gut bacteria species across LVDD grades. **Fig. S5.** Comparisons of associations between metabolites and prevalent (A) and incident (B) LVDD across models. **Fig. S6.** A summary of KOs associated with species. **Fig. S7.** Partial correlation among highlighted LVDD associated species, species associated KOs, and incident LVDD associated metabolites.

** Additional file 2: **Supplementary tables: **Table S1.** Population Characteristics of participants with LVDD information and gut metagenomics data at V2. **Table S2.** Associations of selected gut bacterial species, enzymes, and modules with prevalent LVDD in combined ECHORC-SOL and ECHO-SOL population by adjustment models. **Table S3.** Associations between LVDD-associated species and metabolites. **Table S4.** Associations of gut bacterial species-associated metabolites with prevalent LVDD at V1. **Table S5.** Associations of gut bacterial species-associated metabolites with incident LVDD. **Table S6.** Associations of selected KOs with prevalent LVDD and species. **Table S7.** Relationship among selected KOs, metabolites and species.

## Data Availability

Gut microbiome sequence data in this study are deposited in QIITA (ID 11666). HCHS/SOL has established a process for the scientific community to apply for access to participant data and materials, with such requests reviewed by the project’s steering committee. These policies are described at https://sites.cscc.unc.edu/hchs/. The corresponding authors will accept reasonable requests for data and specimen access, which will be referred to the Steering Committee of the HCHS/SOL project.
