# Supplementary material for: Gut microbiota, blood metabolites, and left ventricular diastolic dysfunction in US Hispanics/Latinos
Source: Microbiome. 2024 May 10;12:85. doi: 10.1186/s40168-024-01797-x (PMC11084054; doi:10.1186/s40168-024-01797-x)
Supplement: Supplementary file 2 — Additional file 1: Supplementary methods: Supplementary Method 1. Study design and population. Supplementary Method 2. Assessment of LVDD. Supplementary Method 3. Gut microbiome profiling. Supplementary Method 4. Metabolome profiling. Supplementary Method 5. Statistical analysis. Supplementary figures: Fig. S1. Overview of the study design and main analyses. Fig. S2. Algorithm for assessing left ventricular diastolic dysfunction (LVDD). Fig. S3. Pooled results of associations between 9 species selected by ANCOM-II and prevalent LVDD in both discovery (ECHORC-SOL) and validation (ECHO-SOL) sets. Fig. S4. Abundances of the identified LVDD-associated gut bacteria species across LVDD grades. Fig. S5. Comparisons of associations between metabolites and prevalent (A) and incident (B) LVDD across models. Fig. S6. A summary of KOs associated with species. Fig. S7. Partial correlation among highlighted LVDD associated species, species associated KOs, and incident LVDD associated metabolites. [file 40168_2024_1797_MOESM1_ESM.docx]

**Supplementary Materials**

**Gut Microbiota, Blood Metabolites and Left Ventricular Diastolic Dysfunction in US Hispanics/Latinos**

Kai Luo, PhD ^1^, Alkis Taryn, MS ^2^, Eun-Hye Moon, MS ^2^, Brandilyn A. Peters, PhD ^1^, Scott D. Solomon, MD ^3^, Martha L. Daviglus, MD ^4^, Mayank M. Kansal, MD ^5^, Bharat Thyagarajan, MD ^6^, Marc D. Gellman, MD ^7^, Jianwen Cai, PhD ^8^, Robert D. Burk, PhD ^1,9-11^, Rob Knight, PhD ^12-15^, Robert C. Kaplan, PhD ^1,16^, Susan Cheng, MD ^17^, Carlos J. Rodriguez, MD ^1,18^, Qibin Qi, PhD ^1,19*^, Bing Yu, PhD^2*^

^1^ Department of Epidemiology and Population Health, Albert Einstein College of Medicine, Bronx, NY 10461, USA

^2^ Department of Epidemiology, Human Genetics and Environmental Sciences, School of Public Health, University of Texas Health Science Center at Houston, Houston, TX 77030, USA

^3^ Brigham and Women’s Hospital, Cardiovascular Medicine, Boston, MA 02115, USA

^4^ Institute for Minority Health Research, University of Illinois Chicago College of Medicine, Chicago, IL 60612, USA

^5^ Clinical Medicine, University of Illinois College of Medicine, Chicago, IL 60612, USA

^6^ Department of Laboratory Medicine & Pathology, University of Minnesota Medical School, Minneapolis, MN 55455, USA

^7^ Department of Psychology, Clinical Research Building, Miller School of Medicine, University of Miami, Miami, FL 33136, USA

^8^ Department of Biostatistics, The University of North Carolina at Chapel Hill, Chapel Hill, NC 27599, USA

^9^ Department of Microbiology and Immunology, Albert Einstein College of Medicine, Bronx, NY 10461, USA

^10^ Department of Obstetrics and Gynecology and Women’s Health, Albert Einstein College of Medicine, Bronx, NY 10461, USA

^11^ Department of Pediatrics, Albert Einstein College of Medicine, Bronx, NY10461, USA

^12^ Center for Microbiome Innovation, University of California, San Diego, La Jolla, CA 92093, USA

^13^ Department of Bioengineering, University of California, San Diego, La Jolla, CA 92093, USA

^14^ Department of Pediatrics, University of California, San Diego, La Jolla, CA 92093, USA

^15^ Department of Computer Science and Engineering, University of California, San Diego, La Jolla, CA 92093, USA

^16^ Public Health Sciences Division, Fred Hutchinson Cancer Research Center, Seattle, WA 98109, USA

^17^ Smidt Heart Institute, Cedars-Sinai Medical Center, Los Angeles, CA 90048, USA

^18^ Department of Medicine, Albert Einstein College of Medicine, Bronx, NY10461, USA

^19^ Department of Nutrition, Harvard T.H. Chan School of Public Health, Boston, MA 02115, USA

**Correspondence**: Dr. **Bing Yu** (bing.yu@uth.tmc.edu) at Department of Epidemiology, Human Genetics and Environmental Sciences, School of Public Health, University of Texas Health Science Center at Houston, 1200 Pressler Street, Houston, TX 77030, USA or Dr. **Qibin Qi** (qibin.qi@einsteinmed.edu) at Department of Epidemiology and Population Health, Albert Einstein College of Medicine, 1300 Morris Park Avenue, Bronx, NY 10461, USA

**List of Contents**

**Supplementary Method1**. Study design and population

**Supplementary Method2**. Assessment of LVDD

**Supplementary Method3**. Gut microbiome profiling

**Supplementary Method4**. Metabolome profiling

**Supplementary Method5**. Statistical analysis

**Fig.S1**. Overview of the study design and main analyses.

**Fig.S2**. Algorithm for assessing left ventricular diastolic dysfunction (LVDD)

**Fig.S3**. Pooled results of associations between 9 species selected by ANCOM-II and prevalent LVDD in both discovery (ECHORC-SOL) and validation (ECHO-SOL) sets

**Fig.S4.** Abundances of the identified LVDD-associated gut bacteria species across LVDD grades.

**Fig.S5**. Comparisons of associations between metabolites and prevalent (A) and incident (B) LVDD across models.

**Fig.S6**. A summary of KOs associated with species.

**Fig.S7**: Partial correlation among highlighted LVDD associated species, species associated KOs, and incident LVDD associated metabolites.

## Supplementary Method1. Study design and population

The HCHS/SOL is the largest ongoing prospective, population-based study of health and diseases in the U.S. Hispanic/Latino population, with 16,415 adults aged 18-74 years recruited at four field centers (Miami, San Diego, Chicago, and the Bronx area of New York) in the United States [1]. The Echocardiographic Study of Latinos (ECHO-SOL) is an ancillary study of the HCHS/SOL designed to characterize the cardiac structure and function in U.S. Hispanic /Latino adults, with 1818 participants aged 45 or over enrolled at baseline from 2011 to 2014[2]; among them, 1643 were followed up with cardiac parameters reexamined at the second clinical visit of the HCHS/SOL (V2) during 2014-2017 [3]. Additionally, the echocardiographic examination was expanded to another independent subset (*N*= 6611) of the HCHS/SOL individuals in the echocardiographic reading centers of field centers (ECHORC-SOL) at V2. The HCHS/SOL collected information on sociodemographic, lifestyle and clinical risk factors at baseline and during the scheduled follow-up visits following standardized study protocols[1]. In the present study, participants with prevalent cardiovascular diseases [e.g., coronary heart diseases (CHD), stroke, heart attack or received heart bypass surgery] were excluded from analyses of LVDD. Informed consent was received from all participants and the current study was approved by the institutional review boards from all participating academic institutes. An overview of study design is shown in **Fig.S1**.

## Supplementary Method2. Assessment of LVDD

A standard transthoracic echocardiography examination, including M-mode, 2D-imaging with harmonics optimizing depth and sector, spectral Doppler, color flow, and tissue Doppler was performed by certified sonographers at each Field Imaging Center through the Philips Ultrasound IE-33 or Sonos 5500/7500 ultrasound imaging platform following the recommendations of American Society of Echocardiography (ASE) [2]. LVDD was defined through a multi-domain system as previously described [2], including (1) pulse-wave Doppler performed in the apical 4 chamber view with the sample volume placed in the mitral valve orifice at the level of the leaflet tips to obtain peak early (E) and late (A) diastolic transmitral inflow velocities; (2) tissue Doppler imaging to acquire mitral early diastolic (e′) annular velocities from the apical 4-chamber view using average values of septal and lateral annular velocities were used here. Value of E/e′ were calculated; and (3) left atrial volume measured in biplane views indexed to body surface area. Following the combination of published ASE and Redfield definitions, LVDD was classified into three grades: grade I (mild), grade II (moderate), and grade III (severe) as shown in **Fig.S2**. Participants who did not meet these criteria for LVDD were defined as grade 0 (i.e., LVDD free group). In main association analysis, we analyzed LVDD as a binary variable with grade I to grade III combined as cases and grade 0 as controls.

## Supplementary Method3. Gut microbiome profiling

During V2, a total of 3,035 participants were enrolled in the Gut Origins of Latino Diabetes (GOLD) ancillary study of the HCHS/SOL [4-6]. Stool samples were collected by participants at home using the Flinders Technology Association (FTA) cards [5, 6]. A shotgun sequencing was performed on the DNA extracted from fecal samples in the Knight laboratory at the University of California San Diego. FASTQ sequence reads were processed using the standard shotgun sequencing pipeline in Qitta and the microbiome taxonomic and functional features were defined through the SHOGUN pipeline [7]. Among the original profiled participants, 2,992 passed the quality control and data filtering with a coverage depth > 100k reads. Among them, 1996 participants received echocardiography assessment, including 1508 in ECHORC-SOL and 488 in ECHO-SOL, and 512 gut bacterial species presented in > 20% of samples were examined. Abundances of species were centered log-ratio (CLR) transformed prior to the analyses (**Fig.S1**).

## Supplementary Method4. Metabolome profiling

Blood metabolomics was conducted using the discoveryHD4 platform at Metabolon (Durham, North Carolina, USA) [8] in 6180 participants of HCHS/SOL at baseline and 814 participants who participated in the GOLD at V2. Combined with information on echocardiography assessment, there were up to 1405 participants included in analyses involving metabolomics data in the present investigation (see **Fig.S1**). We included 669 known metabolites detected in more than 75% of participants. Metabolite levels below detection were imputed by one half of the minimum value. Rank-based inverse normal transformation (INT) was applied to transform levels of metabolites to improve the normality and alleviate the impacts of extreme outlying values on association analyses.

## Supplementary Method5. Statistical analysis

***Associations between gut microbial species and prevalent LVDD***

To identify gut microbial species associated with prevalent LVDD, we first conducted a discovery analysis among 1508 participants (658 prevalent LVDD cases) in the ECHORC-SOL at V2 and then validated the associations of species selected in discovery stage among 488 participants (258 prevalent cases) from the ECHO-SOL at V2 (**Fig.S1**). Herein, we applied the Analysis of Compositions of Microbiomes (ANCOM-II) method using the R package “ANCOMBC” [9] to prioritize gut microbial species that were likely associated with prevalent LVDD in the ECHORC-SOL set (i.e., the discovery set), adjusting for age, sex, study center, usage of antibiotics or probiotics at the time of stool samples collection. Gut bacteria species that had false discovery rate (FDR) < 0.1 with the detection level of ANCOM-II ≥ 0.6 (i.e., the ratio of specific species to at least 60% of the other species detected to be significantly associated with prevalent LVDD) were selected. The associations of species selected by ANCOM-II with prevalent LVDD were then assessed through binary logistic regression in both ECHORC-SOL and ECHO-SOL sets with the same covariate adjustment as in ANCOM-II models. Random-effect meta-analysis was performed to produce pooled associations, and gut microbial species were deemed to be significantly associated with prevalent LVDD if they had significant pooled results (*p* <0.05) and directionally consistent associations in the two analytical sets. The final associations of these species were further assessed in all participants (*N* =1996) in both ECHORC-SOL and ECHO-SOL at V2 using binary logistic regression models adjusted for age, sex, study center, education, annual household income, smoking status, alcohol consumption, physical activity, Alternative Healthy Eating Index 2010 (AHEI2010), and usage of antibiotics or probiotics at the time of stool samples collection (Model1). Herein, study center (“Bronx”, “Chicago”,” Miami”, and “San Diego”), education (“No high school diploma or GED”, “At most a High school diploma or GED”, “High school (or GED) education”, and “University/college education”), family household annual income (“Less than $10,000”, “$10,001-$20,000”, “ $20,001-$40,000” and “More than $40,000”), smoking (“Never”, “former smoker [smoked at least 100 cigarettes in entire life but not now]”, and ”current smoker [smoking daily and at least 100 cigarettes in entire life]”) and alcohol consumption (“Never”, “Former drinker [drunk alcohol before but no now]”, “Current smoker [Presently drinking alcoholic beverages]”) status were modeled as categorical variables. Physical activity was modeled as a numeric variable (MET-min/day) which was the total amount of time spent on vigorous-intensity, moderate-intensity, walk or bicycle, vigorous-intensity sports, fitness or recreational (leisure), and moderate-intensity sports, fitness or recreational (leisure) activities per week. AHEI2010 was an index assessing the overall dietary quality with values ranging from 0 to 110 [10]. There were 11 components included in AHEI2010, 1) vegetables without potatoes, servings/day; 2) whole Fruit (i.e. does not include fruit juice), servings/day; 3) whole grains, servings/day; 4) sugar sweetened beverages and fruit juice, servings/day; 5) nuts and legumes, servings/day; 6) red/processed meat, servings/day; 7) trans Fat, % energy; 8) long-chain (n-3) fats (EPA+DHA), mg/day; 9) polyunsaturated fatty acids (PUFA), % energy; 10) sodium, mg/day; 11) alcohol, drinks/day. The frequencies of these covariates and their definitions were also listed in **Table S1**.

To test the robustness of associations between selected gut microbial species and prevalent LVDD, we additionally considered two multivariable adjustment models: Model1 plus body mass index (BMI) and systolic blood pressure (SBP) (Model2), and Model1 plus BMI, SBP, and use of antidiabetic, antihypertensive, and lipid lowering medications (Model3).

***Metabolomics signatures of prevalent LVDD-associated bacterial species and their associations with LVDD***

Given that the blood metabolome is a comprehensive readout of both gut and host metabolism[11], the alterations in circulating metabolites could explain the relationship between gut microbiota and LVDD. We thus first conducted metabolome wide association analysis (MWAA) to identify metabolites signatures of prevalent LVDD-associated microbial species. Herein, multiple linear regressions adjusted for covariates in Model1 were conducted among 804 participants who had concurrent microbiome and metabolome data at V2. The associations of species-associated metabolites with prevalent LVDD were then examined among 1405 participants (695 of whom had prevalent cases) enrolled in the ECHO-SOL at V1, using a binary logistic regression model adjusted for the same set of covariates (except for the antibiotics or probiotics usage) included in the above MWAA. FDR was controlled at 0.1. In addition, we conducted a prospective analysis to assess the relationship between these prevalent LVDD-associated metabolites and incident LVDD over a mean follow-up of 4.3 years [3] among 594 participants (212 had incident cases) who were free of LVDD at baseline. In this incident analysis, a modified Poisson regression model [12] was used given that the incident LVDD is a common binary outcome (proportion of incident cases > 30%) in our study population. The robustness of associations between species-associated metabolites and prevalent and incident LVDD was assessed through additional adjustments in Model2 and Model3 as mentioned before.

Given the lack of prospective data for gut microbiome and incident LVDD analysis, we conducted “proxy association” analyses [13] by using identified bacterial-associated metabolites as proxies to indirectly assess the relationship between gut bacteria and incident LVDD. In brief, the effect estimates for the associations of metabolites with bacteria species and incident LVDD were standardized into Z-scores (i.e., the ratios of beta coefficients to their respective standard errors). A spearman correlation was then conducted to link Z-scores for metabolites-species associations with those for metabolites-LVDD associations, in which a correlation p-value < 0.05 indicating a significant proxy association between gut bacterial species and incident LVDD. Only metabolites significantly associated with incident LVDD were included in this proxy-association analysis. Z-scores were used to increase the comparability between effect estimates with varied units. ***Prediction of incident LVDD utilizing identified gut bacterial species associated metabolites***

To assess the relative importance of identified metabolites and traditional risk factors in predicting incident LVDD, we constructed an elastic net regression (ENR) model among the abovementioned 597 participants who were free of LVDD at baseline. In this ENR analysis, input features subjected to feature selection with penalization included the identified 21 bacterial species associated metabolites and 6 traditional risk factors of HF (age, BMI, SBP, smoking, drinking, low low-density lipoprotein cholesterol (LDL-C), and diabetes) with a set of covariates (study filed center, gender, education, annual household income, physical activity and AHEI2010) being adjusted for without penalization. Tuning parameters were selected via a 10 × 10-fold nested cross-validation (nestedcv), a method designed to maximize the use of the whole dataset for testing overall accuracy, while maintaining the split between training and testing to avoid the overfitting [14]. This ENR analysis was performed using the R package “nestedcv”. The area under the curve (AUC) was calculated to performance of prediction model in reclassification of incident LVDD. The 95%CIs of AUC were estimated via bootstrapping with 10,000 iterations using the R package “pROC”. The relative importance of selected features in this ENR analysis was assessed via the SHapley Additive exPlanations (SHAP), a model-agnostic index assessing the impacts of input features on the outcome [15]. Specifically, the SHAP values were calculated via the R package “fastshap”.


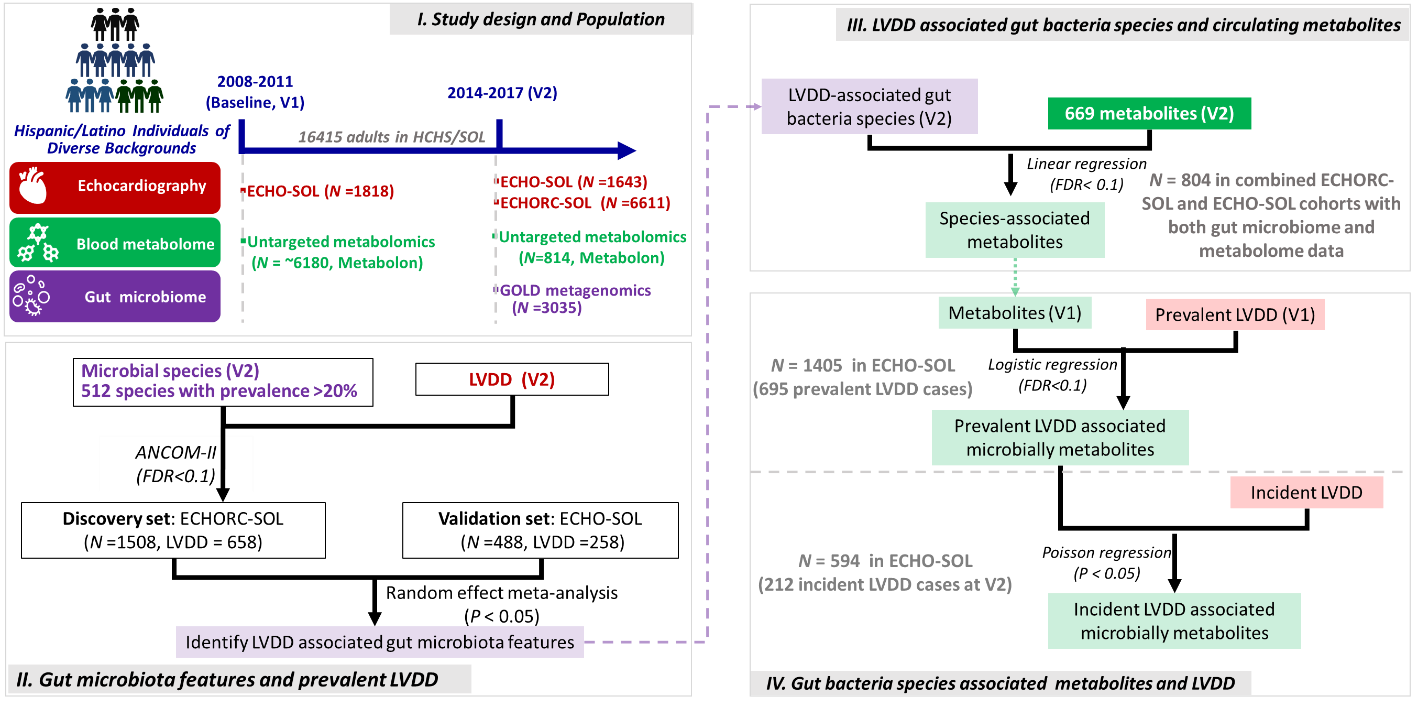


**Fig.S1. Overview of the study design and main analyses.** The baseline recruitment of ECHO-SOL was conducted from 2011-2014.


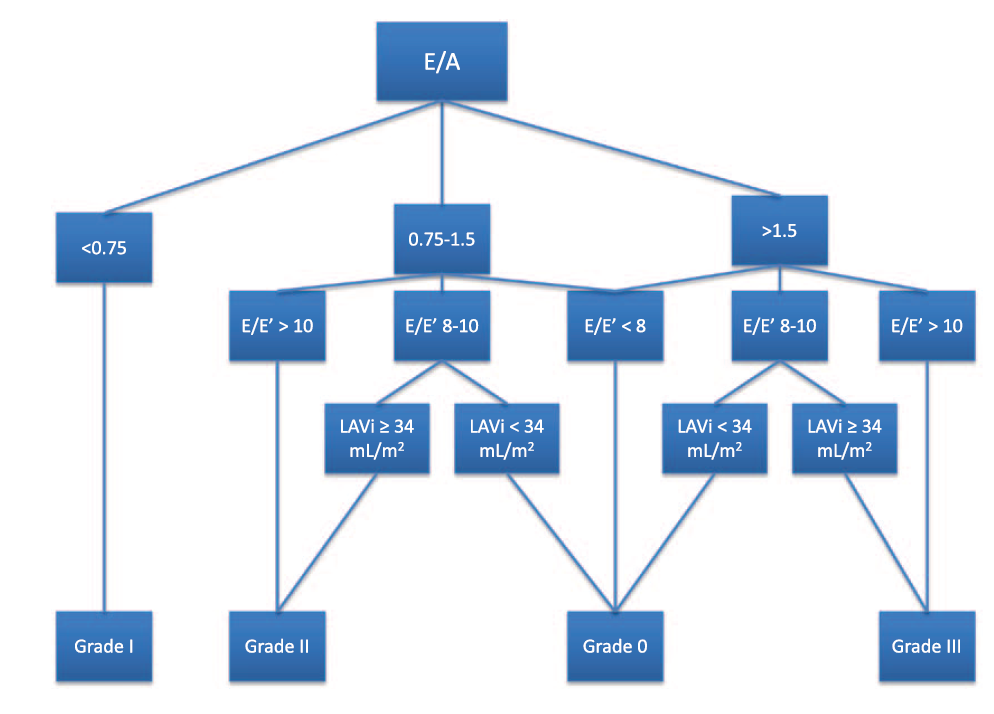


**Fig.S2. Algorithm for assessing left ventricular diastolic dysfunction (LVDD)** via echocardiography based on the Redfield and American Society of Echocardiography (ASE) criteria as previously described [2]. Abbreviations: E/A: the ratio of peak early (E) and late (A) diastolic transmitral inflow velocities; E/E’: the ratio of early to mitral early diastolic (e’) annular velocities; LAVi: left atrial volume index.


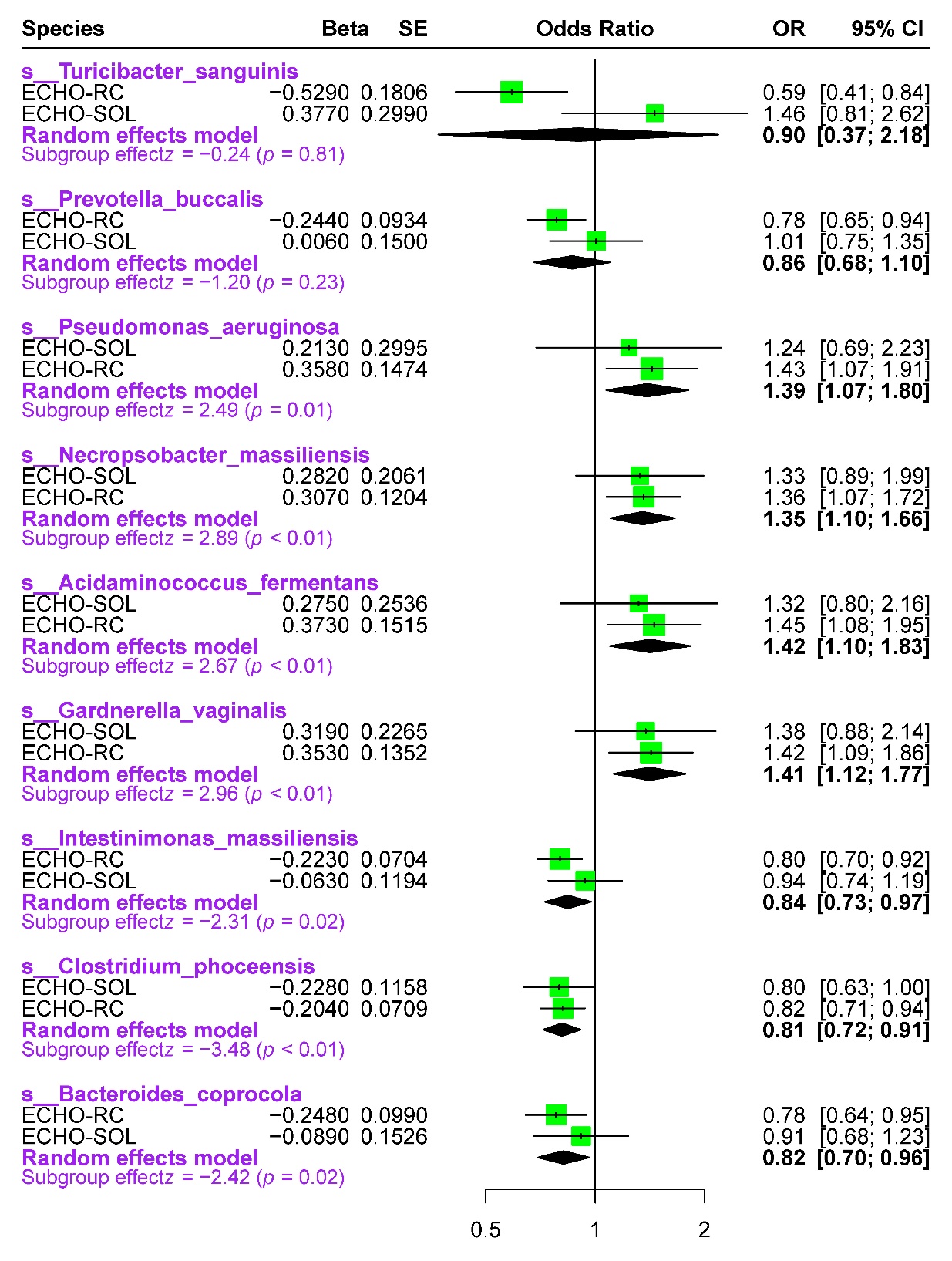


**Fig.S3. Pooled results of associations between 9 species selected by ANCOM-II and prevalent LVDD in both discovery (ECHORC-SOL) and validation (ECHO-SOL) sets**. Associations were assessed through binary logistic regression while adjusting for age, sex, study center, and use of antibiotics or probiotics at the time of stool sample collection. The abundances of included species were central log ratio transferred. Random effect meta-analysis was used to pool associations found in discovery and validation sets.


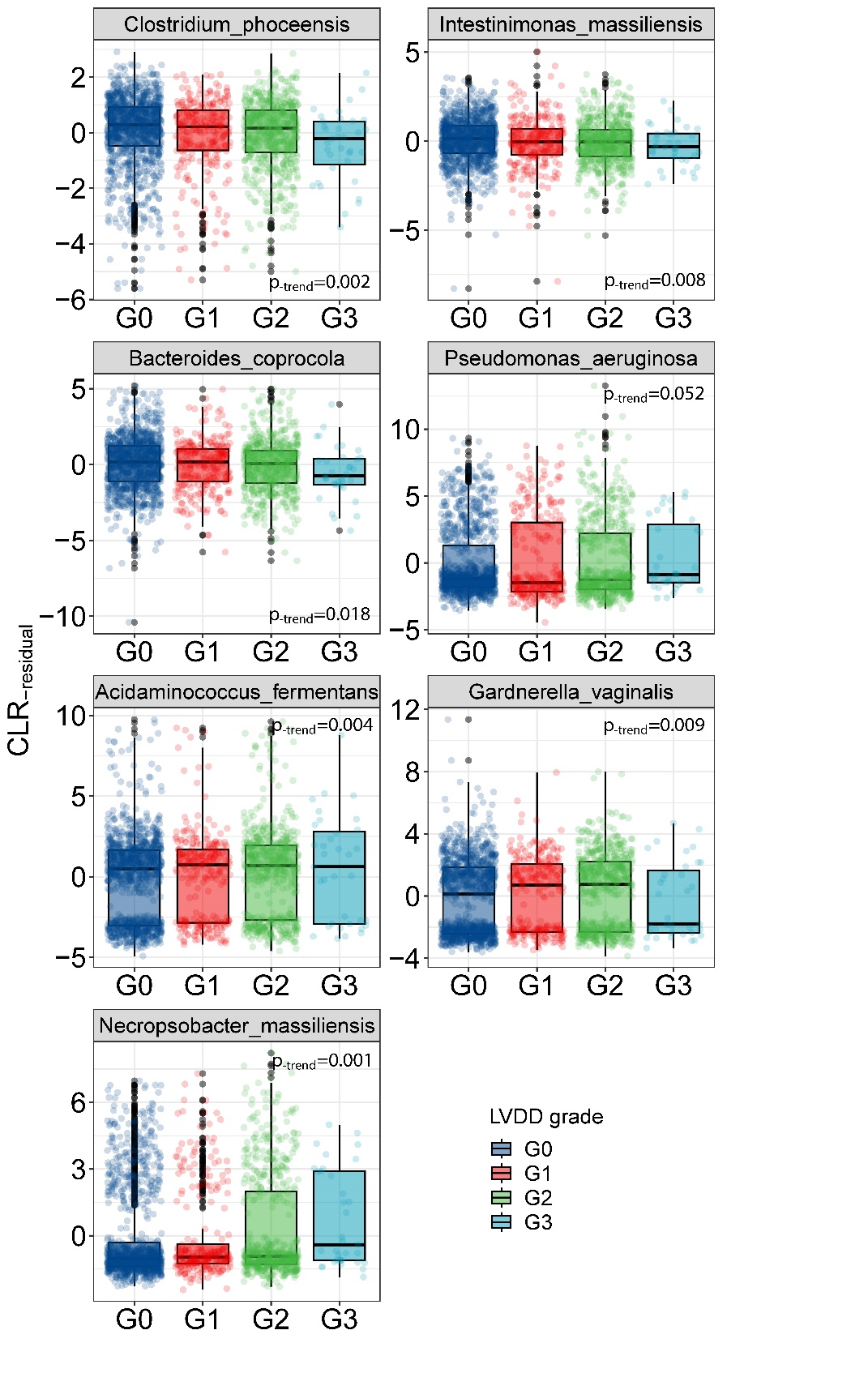


**Fig.S4. Abundances of identified LVDD-associated gut bacteria species across LVDD grades.** Abundance values of Y-axis were shown as residuals of central log-ratio transformed abundances regressed on age, sex, study center, education, annual income, smoking status, alcohol consumption, physical activity (METs), AHEI2010, and use of antibiotics or probiotics at the time of stool sample collection. *P*-trend was derived in regression models with the LVDD grades modeled as an ordinal variable.


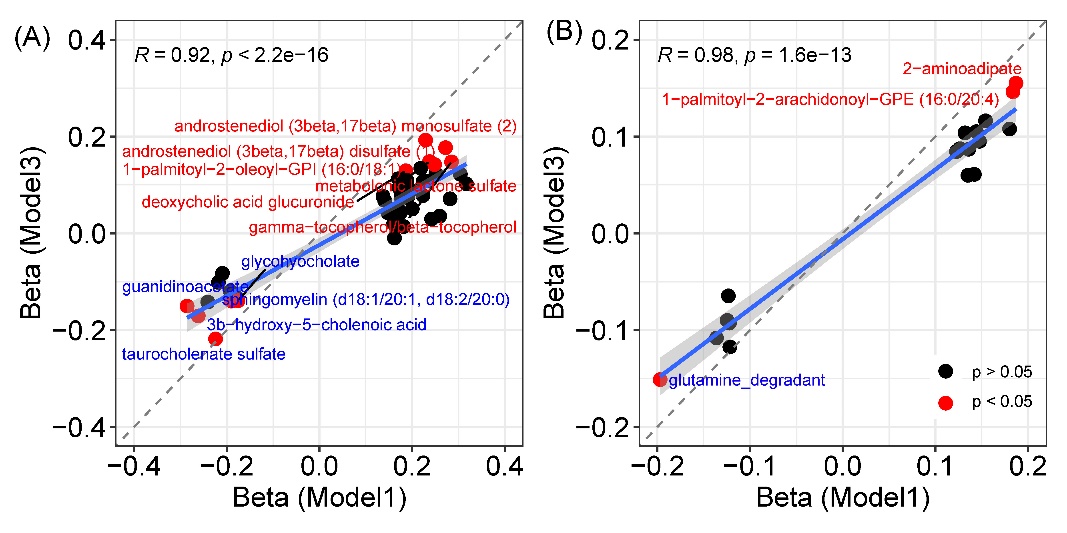


**Fig.S5. Comparisons of associations between metabolites and prevalent (A) and incident (B) LVDD across models.** Logistic regression model and modified Poisson regression model were applied. Data were shown as regression coefficient (Beta). Model1: adjusted for baseline age, gender, study center, education, income, cigarette use, alcohol use, physical activity (METs), and AHEI2010; Model3: Model1 plus baseline BMI, SBP, the use of antidiabetic, lipids lowering and antihypertensive medications.


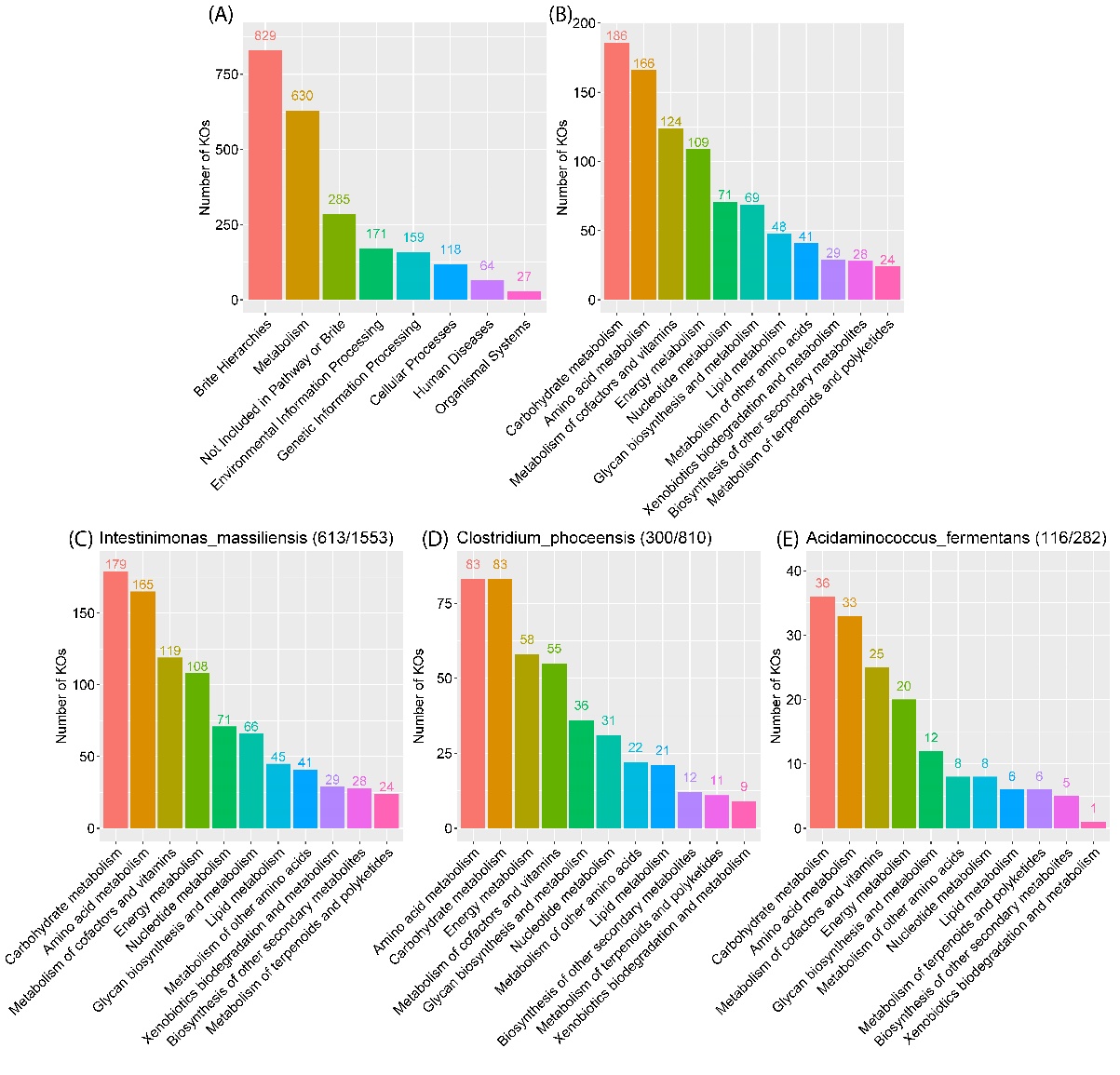


**Fig.S6**. A summary of KOs associated with species. (A) the distribution of KOs significantly associated with at least of 7 species by KO pathway level A. (B) The distribution of KOs significantly associated with at least of 7 species (FDR < 0.1) and involved in metabolism pathway. (C) to (E) Metabolism sub-pathway specific distribution of KOs that were respectively associated *Intestinimonas_massiliensis*, *Clostridium_phoceensis*, and *Acidaminococcus_fermentans*. Only data of these species that have significant proxy associations with prevalent LVDD were shown here.


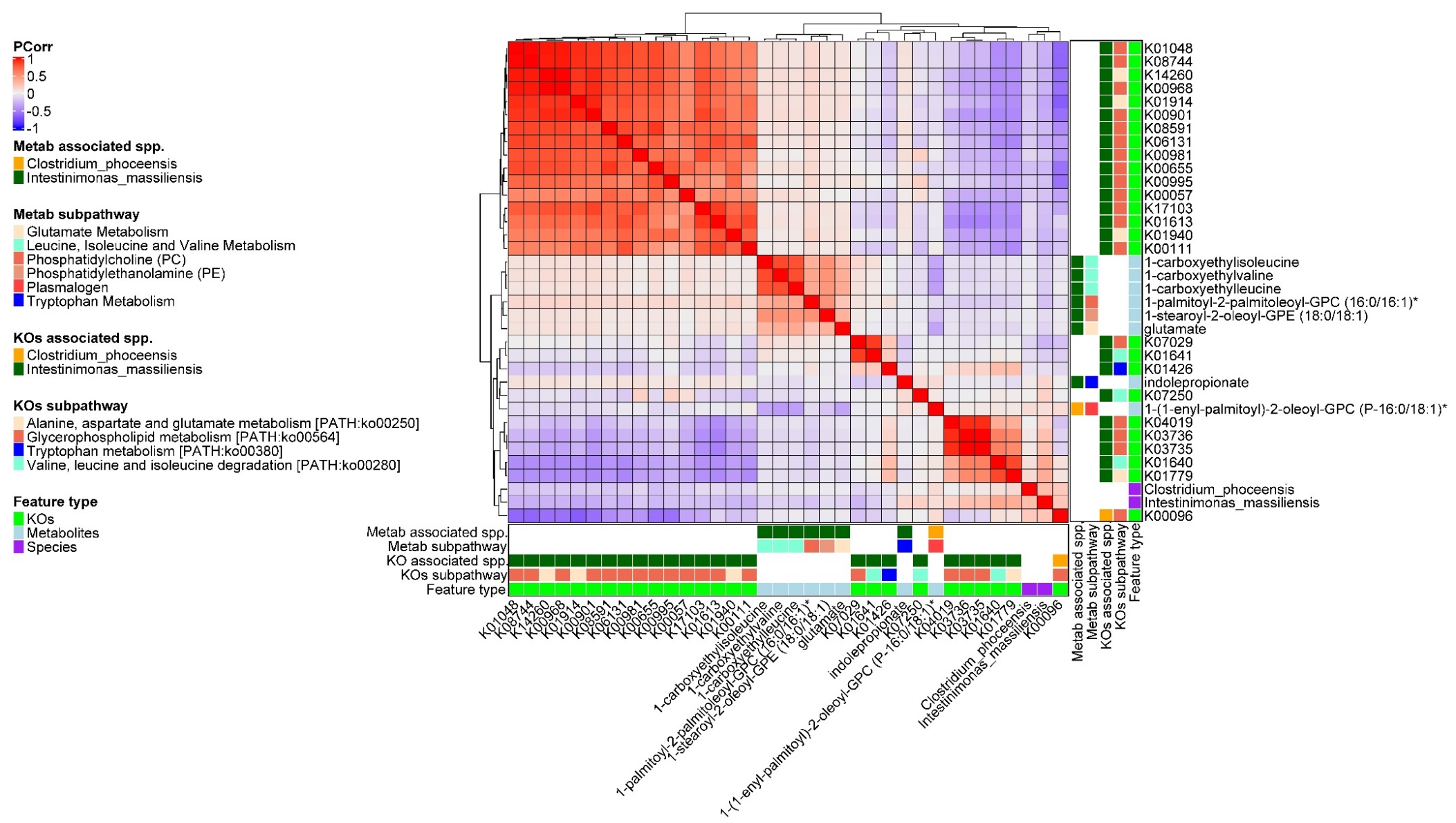


**Fig.S7**: Partial correlation among highlighted LVDD associated species, species associated KOs, and incident LVDD associated metabolites. Covariates adjusted for included age, gender, study center, education, income, cigarette use, alcohol use, physical activity (METs), AHEI2010, and use of antibiotics or probiotics at time of stool sample collection.

**References:**

1. Pirzada A, Cai J, Heiss G, Sotres-Alvarez D, Gallo LC, Youngblood ME, Aviles-Santa ML, Gonzalez HM, Isasi CR, Kaplan R, et al: **Evolving Science on Cardiovascular Disease Among Hispanic/Latino Adults: JACC International.** *J Am Coll Cardiol* 2023, **81:**1505-1520.

2. Mehta H, Armstrong A, Swett K, Shah SJ, Allison MA, Hurwitz B, Bangdiwala S, Dadhania R, Kitzman DW, Arguelles W, et al: **Burden of Systolic and Diastolic Left Ventricular Dysfunction Among Hispanics in the United States: Insights From the Echocardiographic Study of Latinos.** *Circ Heart Fail* 2016, **9:**e002733.

3. Kuno T, Vasquez N, April-Sanders AK, Swett K, Kizer JR, Thyagarajan B, Talavera GA, Ponce SG, Shook-Sa BE, Penedo FJ, et al: **Pre-Heart Failure Longitudinal Change in a Hispanic/Latino Population-Based Study: Insights From the Echocardiographic Study of Latinos.** *JACC Heart Fail* 2023.

4. Usyk M, Peters BA, Karthikeyan S, McDonald D, Sollecito CC, Vazquez-Baeza Y, Shaffer JP, Gellman MD, Talavera GA, Daviglus ML, et al: **Comprehensive evaluation of shotgun metagenomics, amplicon sequencing, and harmonization of these platforms for epidemiological studies.** *Cell Rep Methods* 2023, **3:**100391.

5. Kaplan RC, Wang Z, Usyk M, Sotres-Alvarez D, Daviglus ML, Schneiderman N, Talavera GA, Gellman MD, Thyagarajan B, Moon JY, et al: **Gut microbiome composition in the Hispanic Community Health Study/Study of Latinos is shaped by geographic relocation, environmental factors, and obesity.** *Genome Biol* 2019, **20:**219.

6. Wang Z, Usyk M, Vazquez-Baeza Y, Chen GC, Isasi CR, Williams-Nguyen JS, Hua S, McDonald D, Thyagarajan B, Daviglus ML, et al: **Microbial co-occurrence complicates associations of gut microbiome with US immigration, dietary intake and obesity.** *Genome Biol* 2021, **22:**336.

7. Hillmann B, Al-Ghalith GA, Shields-Cutler RR, Zhu Q, Knight R, Knights D: **SHOGUN: a modular, accurate and scalable framework for microbiome quantification.** *Bioinformatics* 2020, **36:**4088-4090.

8. Feofanova EV, Chen H, Dai Y, Jia P, Grove ML, Morrison AC, Qi Q, Daviglus M, Cai J, North KE, et al: **A Genome-wide Association Study Discovers 46 Loci of the Human Metabolome in the Hispanic Community Health Study/Study of Latinos.** *Am J Hum Genet* 2020, **107:**849-863.

9. Lin H, Das Peddada S: **Analysis of compositions of microbiomes with bias correction.** *Nature Communications* 2020, **11**.

10. Chiuve SE, Fung TT, Rimm EB, Hu FB, McCullough ML, Wang M, Stampfer MJ, Willett WC: **Alternative dietary indices both strongly predict risk of chronic disease.** *J Nutr* 2012, **142:**1009-1018.

11. Bar N, Korem T, Weissbrod O, Zeevi D, Rothschild D, Leviatan S, Kosower N, Lotan-Pompan M, Weinberger A, Le Roy CI, et al: **A reference map of potential determinants for the human serum metabolome.** *Nature* 2020, **588:**135-140.

12. Zou G: **A modified poisson regression approach to prospective studies with binary data.** *Am J Epidemiol* 2004, **159:**702-706.

13. Amin N, Liu J, Bonnechere B, MahmoudianDehkordi S, Arnold M, Batra R, Chiou YJ, Fernandes M, Ikram MA, Kraaij R, et al: **Interplay of Metabolome and Gut Microbiome in Individuals With Major Depressive Disorder vs Control Individuals.** *JAMA Psychiatry* 2023, **80:**597-609.

14. Lewis MJ, Spiliopoulou A, Goldmann K, Pitzalis C, McKeigue P, Barnes MR: **nestedcv: an R package for fast implementation of nested cross-validation with embedded feature selection designed for transcriptomics and high-dimensional data.** *Bioinformatics Advances* 2023, **3:**vbad048.

15. Lundberg SM, Lee S-I: **A unified approach to interpreting model predictions.** *Advances in neural information processing systems* 2017, **30**.
